# Increased mutation efficiency of CRISPR/Cas9 genome editing in banana by optimized construct

**DOI:** 10.7717/peerj.12664

**Published:** 2022-01-05

**Authors:** Sen Zhang, Shaoping Wu, Chunhua Hu, Qiaosong Yang, Tao Dong, Ou Sheng, Guiming Deng, Weidi He, Tongxin Dou, Chunyu Li, Chenkang Sun, Ganjun Yi, Fangcheng Bi

**Affiliations:** 1 Key Laboratory of South Subtropical Fruit Biology and Genetic Resource Utilization (Ministry of Agriculture and Rural Affairs), Guangdong Key Laboratory of Tropical and Subtropical Fruit Tree Research, Institute of Fruit Tree Research, Guangdong Academy of Agricultural Sciences, Guangzhou, Guangdong, China; 2 College of Horticulture and Forestry Sciences, Huazhong Agricultural University, Wuhan, Hubei, China; 3 College of Life Sciences, Zhaoqing University, Zhaoqing, Guangdong, China; 4 College of Life Sciences, South China Agricultural University, Guangzhou, Guangdong

**Keywords:** CRISPR/Cas9, Genome editing, Banana, Vector optimization

## Abstract

The CRISPR/Cas9-mediated genome editing system has been used extensively to engineer targeted mutations in a wide variety of species. Its application in banana, however, has been hindered because of the species’ triploid nature and low genome editing efficiency. This has delayed the development of a DNA-free genome editing approach. In this study, we reported that the endogenous U6 promoter and banana codon-optimized Cas9 apparently increased mutation frequency in banana, and we generated a method to validate the mutation efficiency of the CRISPR/Cas9-mediated genome editing system based on transient expression in protoplasts. The activity of the MaU6c promoter was approximately four times higher than that of the OsU6a promoter in banana protoplasts. The application of this promoter and banana codon-optimized Cas9 in CRISPR/Cas9 cassette resulted in a fourfold increase in mutation efficiency compared with the previous CRISPR/Cas9 cassette for banana. Our results indicated that the optimized CRISPR/Cas9 system was effective for mutating targeted genes in banana and thus will improve the applications for basic functional genomics. These findings are relevant to future germplasm improvement and provide a foundation for developing DNA-free genome editing technology in banana.

## Introduction

The *Streptococcus*-derived CRISPR/Cas9 system is an effective site-specific genome editing tool (Mali et al., 2013). One component of this system, the CRISPR-associated protein 9 (Cas9) endonuclease, can produce double-stranded DNA (dsDNA) breaks on target sites guided by another component, a single-guide RNA (sgRNA) (Jinek et al., 2012; Cong et al., 2013). The error-prone nonhomologous end joining (NHEJ) repair for DSBs can introduce deletions and insertions at the broken ends, generating targeted genome modifications (Budman & Chu, 2005; Gong et al., 2005). The homology-directed repair (HDR) for DSBs can produce mutations or insertions of an entire gene or genetic elements at the target site using different template sequences (Zha, Boboila & Alt, 2009). Compared with previously discovered Zinc finger nucleases (ZFNs) and Transcription activator-like Effector Nucleases (TALENs) technology (Kim, Cha & Chandrasegaran, 1996; Miller et al., 2011), the CRISPRCas9 system is easier to operate and much more cost-effective for targeting different genome sites. It is becoming the most potent tool for gene knockouts, base replacement, and regulation of gene transcription in plants (Razzaq et al., 2019). Moreover, in addition to basic functional genomics research, this system has been used for targeted gene modifications or molecular breeding in various fruit crops, including tomato (Yang et al., 2017; Yu et al., 2017; Li et al., 2018), sweet orange (Jia & Wang, 2014; Zhang et al., 2017), Duncan grapefruit (Jia et al., 2016; Jia et al., 2017; Peng et al., 2017), apple (Malnoy et al., 2016; Nishitani et al., 2016), grapevine (Ren et al., 2016; Wang et al., 2018a; Wang et al., 2018b), watermelon (Tian et al., 2017), kiwifruit (Wang et al., 2018a; Wang et al., 2018b), cucumber (Chandrasekaran et al., 2016; Hu et al., 2017a; Hu et al., 2017b), strawberry (Zhou, Wang & Liu, 2018; Feng et al., 2019), and banana (Hu et al., 2017a; Hu et al., 2017b; (Kaur et al., 2018; Naim et al., 2018; Tripathi et al., 2019; Ntui, Tripathi & Tripathi, 2020).

Banana is ranked fourth after to rice, wheat, and maize as the most important food crop in developing countries according to the United Nations Food and Agriculture Organization (FAO). Banana production faces various challenges, such as postharvest losses and biotic and abiotic stresses. The most effective way to overcome these problems is the development of resistant varieties. The CRISPR/Cas9-based genome editing system has become the best tool for banana molecular breeding (Tripathi, Ntui & Tripathi, 2020). Recently, Tripathi et al. (2019) successfully inactivated the eBSV integrated into the genome of plantain by editing the virus sequences to tackle the major challenge to banana breeding (Tripathi et al., 2019).

The majority of edible cultivated bananas originated from intraspecific or interspecific hybridization between wild diploid *M. acuminata* (A-genome) and *M. balbisiana* (B-genome) species (Wang et al., 2019). There are various genotypes of cultivated edible bananas, including diploids (AA, BB, and AB), triploids (AAA, AAB, and ABB), and tetraploids (AAAB, AABB, and ABBB) that derived from different combinations of A- and B-genomes (Simmonds & Shepherd, 1955). The triploid genotype variants are the predominant cultivated varieties in the world. The complexity of the banana genomes or genotypes, including heterozygosity and polyploidy, results in more allelic or copies of genes that need to be edited for the expected phenotype in applying genome editing. The editing efficiency of the CRISPR/Cas9 system varies with the species due to different genome structural factors, such as genome size and GC content (Bortesi et al., 2016). Further work for optimizing the bacterial-derived CRISPR/Cas9 system is required for effective application in different organisms. At present, the main methods for increasing the mutation efficiency of the CRISPR/Cas9 system are through improving Cas9 or gRNA expression using different strategies (Zhu et al., 2016; Mao, Botella & Zhu, 2017; LeBlanc et al., 2018). Increased mutation frequency of the CRISPR/Cas9 system by using different constitutive, tissue-specific, or germline-specific promoters to drive Cas9 expression has been demonstrated in plants (Wang et al., 2015; Yan et al., 2015; Eid, Ali & Mahfouz, 2016; Mao et al., 2016; Feng et al., 2018; Ordon et al., 2020). Moreover, using plant or species codon-optimized Cas9 can enhance Cas9 activity and increase mutation frequency (Bortesi & Fischer, 2015; Zhu et al., 2016). LeBlanc et al. (2018) reported that heat treatment could apparently improve Cas9 activity and result in increased efficiency of targeted mutagenesis induced by CRISPR/Cas9 in plants. Another factor employed to enhance the mutagenic efficiency of CRISPR/Cas9 is improving the expression of gRNA (Ng & Dean, 2017). Although OsU6 or U3 promoters and AtU6 or AtU3 promoters can efficiently drive gRNA expression in monocots and dicots, respectively (Ma et al., 2015), researchers have found that endogenous U6 or U3 promoters can apparently improve sgRNA expression and result in increased efficiency of targeted mutagenesis (Feng et al., 2018; Long et al., 2018; Bernard et al., 2019).

In this study, we optimized the CRISPR/Cas9 system in banana by increasing the expression of sgRNA and Cas9 proteins. Our results showed that endogenous promoters significantly increased the expression of sgRNA. Moreover, the newly configured CRISPR/Cas9 system increased the mutagenesis efficiency in banana protoplasts, and this was confirmed by deep amplicon sequencing. In addition, we developed a frameshift in the fused-LUC (fLUC) system to examine the genome editing efficiency and for screening the effectiveness of the sgRNA. Our data confirmed that the optimized CRISPR/Cas9 system could be applied for robust targeted genome editing in banana.

## Material and Methods

### Banana protoplasts isolation

The banana embryogenic cell suspensions (ECS) for protoplasts preparation were induced from male flower buds of *Musa acuminat* a (AAA group, cv. Brazilian) cultivated at the Institute of Fruit Tree Research, Guangdong Academy of Agricultural Sciences, Guangzhou, P. R. of China. The Cavendish Banana variety that we used is the main cultivar in China. The ECS was maintained for several years and subcultured every two weeks with M2 media (Dong et al., 2020). For protoplasts isolation, ECS that were subcultured for 10 days were collected and re-suspended in 10 mL of enzymatic hydrolysate (3.0% cellulose R-10, 1% segregation enzyme R-10, 0.2% pectinase Y-23, 15.2 g.l^−1^ KCl, 7.8 g.l^−1^ CaCl_2_, 100 mg. l^−1^ MES, 10% mannitol, pH 5.7) and were gently shaken at 50 rpm for 6–8 h. The released protoplasts were passed through Miarcloth (EMD Millipore, Billerica, MA, USA) and collected after washing with W5 media (154 mM NaCl, 125 mM CaCl_2_, 5 mM KCl, and 2 mM MES adjusted to pH 5.7 with KOH).

### Vector construction

For obtain the promtoer sequences of U6 gene of banana, we performed a BLAST search against banana genome with U6a sequence of Oryza sativa L (Ma et al., 2015) as query. Different length sequences before transcription start site of U6 small RNA was considered as candidate promoter. To check the activity of the promoter, we introduced the promoter sequences of interest into a pGreen II 0800-LUC vector to obtain pGreen II 0800-Promoters for dual-luciferase reporter assays. All promoter sequences and sgRNA sequences used in this study are listed in Supplementary File 2. The banana codon-optimized Cas9 (Supplementary File 1) or Cas9 open reading frame with nuclear localization signals was synthetically produced and cloned into the vector pCAMBIA1300 with *Eco* R I/*Kpn* I and *Eco* R I/*Xba* I to obtain pCAMBIA1300-opCas9 or pCAMBIA1300-Cas9, respectively. The pUbi was cloned into pCAMBIA1300-opCas9 or pCAMBIA1300-Cas9 with *Eco* R I to obtain pCAMBIA1300-pUbi::opCas9 or pCAMBIA1300-pUbi::Cas9 constructs using a ClonExpress^®^ II One Step Cloning Kit (Vazyme Biotech Co., Ltd, C112). To create the gRNA expression cassette, the sgRNA scaffold and sgRNA promoter with the sgRNA sequence were amplified separately and cloned into pCAMBIA1300-pUbi/::opCas9 or pCAMBIA1300-pUbi::Cas9 vectors using a ClonExpress^®^ II MultiS One Step Cloning Kit (Vazyme Biotech Co., Ltd, C113) with the *Bsa* I site. The sgRNA scaffold was amplified from pYLsgRNA-OsU6a vector (Ma et al., 2015) and all MaPDS target sites (Wu et al., 2020) used in this study were introduced into vectors by synthetic primers. To construct the fused luciferase vector, a 24 bp fragment that was targeted by sgRNA-MaPDS was inserted after the luciferase start codon in a pGreen II 0800-LUC vector to produce fused and functional luciferase. All primers used in this study are listed in Supplementary File 3.

### Protoplasts transformation

The protoplasts transformation was performed as described previously (Wu et al., 2020). For each sample, 20 µg plasmids (10 µg plasmid for each if for co-transformation), 200 *μ*L protoplasts (2 ×10^6^–2 ×10^7^.ml^−1^), and 250 *μ*L 50% PEG 4000 (Sigma-Aldrich, Shanghai, China) were gently mixed in a 2-mL centrifuge tube and incubated for 30 min in darkness. Then, 900 *μ*L W5 solution was added for stopping transfection. After centrifugation at 100 g for 3 min and removing the supernatant, the cells were resuspended with one mL W5 media and incubated for 4–5 days in darkness at room temperature.

### Dual-luciferase reporter assay

The whole assay was performed according to the technical manual of the dual-luciferase reporter assay system (Promega, Madison, WI, USA). The dual luciferase reporter gene usually used firefly luciferase (LUC) as reporter gene and renilla luciferase (REN) as internal reference gene. After 4–5 days of incubation, the activities of Firefly Luciferase and Renilla Luciferase of collected protoplasts were measured by a Multimode Microplate Reader (Tecan, Männedorf, Switzerland). We used the LUC/REN ratio to indicate the relative activity of each of the cloned promoters and repeated the experiments at least three times.

### Deep amplicon sequencing

Five days after transformation, protoplasts were collected to extract genomic DNA for deep sequencing with a DNA extraction kit (TIANGEN BIOTECH CO., LTD, Beijing, China). After two-round PCR amplification using site-specific primers (Supplementary File 3), an equal amount (200 ng) of PCR products after gel extractionfrom each sample were mixed as a pool for Illumina sequencing at Sangon Biotech Co., Ltd. (Shanghai, China). The length of second-round PCR products was approximately 200 bp. The forward and reverse barcodes were added to the ends of the specific primers in the second round PCR for library construction. Indels, including insertions and deletions, occurring at 3–4 bp upstream PAM site were considered as mutations.

## Results

### Identification of U6 promoters in banana

**Figure 1:**
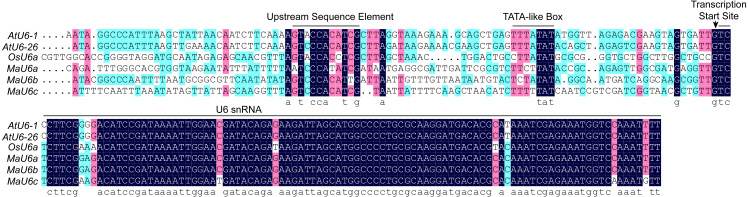
Multiple alignments of banana, rice, and Arabidopsis U6 gene and promoter sequences. Upstream Sequence Elements (USE), the TATA-boxes, and the U6 small nuclear (snRNA) transcripts are marked with black bars. The transcription start sites are indicated with an arrow.

For CRISPR/Cas9 system in monocots, rice U6 or U3 promoters are commonly used to drive the expression of sgRNAs because of their capacity to obtain high expression levels. Recently, we successfully established a CRISPR/Cas9 genome editing system in banana using a vector that included the rice U6a promoter (Hu et al., 2017a; Hu et al., 2017b). To optimize the CRISPR/Cas9 system in banana, we performed a BLAST search against the banana genome with OsU6a as a query, and three homologous sequences with high homology were returned. The three sequences were regarded as MaU6a, MaU6b, and MaU6c (Fig. 1A). The U6 snRNA sequences between rice and banana were highly conserved, but their promoter region sequences were different, except for the upstream sequence element (USE) and the TATA-like box sequences (Fig. 1), suggesting that they might have unequal activity in driving sgRNA expression in the same plant.

### Determination of the activity of endogenous MaU6 promoter

**Figure 2:**
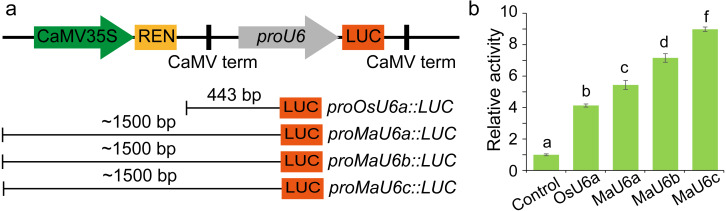
Determination of the activity of banana endogenous promoter. (A) Representation of LUC expression constructs using diferent promoter fragments, (B) Examination of promoter activity for driving the expression of LUC. Approximately 1,500 bp promoters were used to drive the expression of LUC. Bars mean standard deviations for the three independent replicates. Different letters indicate significant differences assessed using one-way analysis variance (ANOVA), followed Fisher’s protected least significant difference test ( *P* < 0.05).

**Figure 3:**
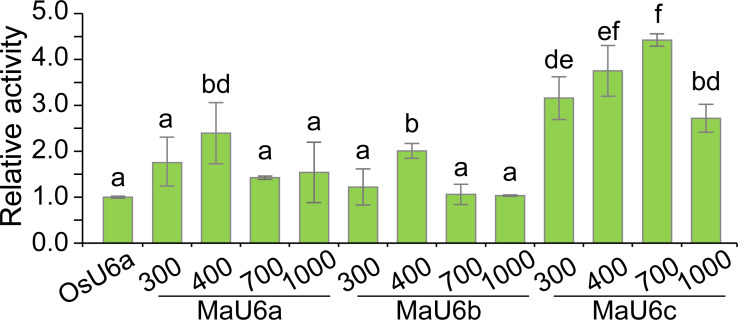
Activity analysis of different sizes of U6 promoters for driving LUC expression. Vertical bars represent standard deviations of the four independent replicates. Different letters indicate significant differences assessed using one-way analysis variance (ANOVA), followed Fisher’s protected least significant difference test ( *P* < 0.05).

To compare the activities of these promoters, we introduced approximately 1500 bp sequences upstream from the transcription initiation sites of three promoters into a pGreen II 0800-LUC vector with *Spe* I upstream of the luciferase reporter gene (LUC). We cloned a 443 bp OsU6a promoter sequence that has been used in rice genome editing (Ma et al., 2015) into pGreen II 0800-LUC and considered it to be a control (Fig. 2). We performed transient transformation experiments in banana protoplasts by PEG-mediated transformation (Wu et al., 2020). We used the dual-luciferase reporter assay to analyze the promoter activity. The results indicated that all examined promoters could drive luciferase expression, and three banana U6 promoters exhibited higher activity than that of OsU6a in protoplasts isolated from embryogenic cell suspensions of banana (Fig. 2). To further test the activity of promoter fragments of different lengths for each banana U6 promoter, we cloned 1000 bp, 700 bp, 400 bp, and 300 bp promoter sequences into the upstream region of the luciferase reporter gene in the pGreen II 0800-LUC vector. The results showed that the activity of most examined MaU6 promoters was higher than that of pOsU6a except for 300-, 700- and 1000-bp promoters of MaU6a and MaU6b in banana (Fig. 3). The promoter MaU6c (700 bp) had the highest activity, generating four times the activity of the OsU6a promoter, suggesting that it might be the best choice for driving gRNA expression in banana. The pMaU6a (400 bp) and pMaU6b (400 bp) also can be used to drive gRNA expression in CRISPR/Cas9-mediated gene editing of banana, especially for co-editing with different target sites. Finally, we chose the fragment pMaU6c (700 bp) to examine its efficiency in CRISPR/Cas9-mediated gene editing using banana protoplasts.

### Examination of genome editing efficiency of the optimized CRISPR/Cas9 system

**Figure 4:**
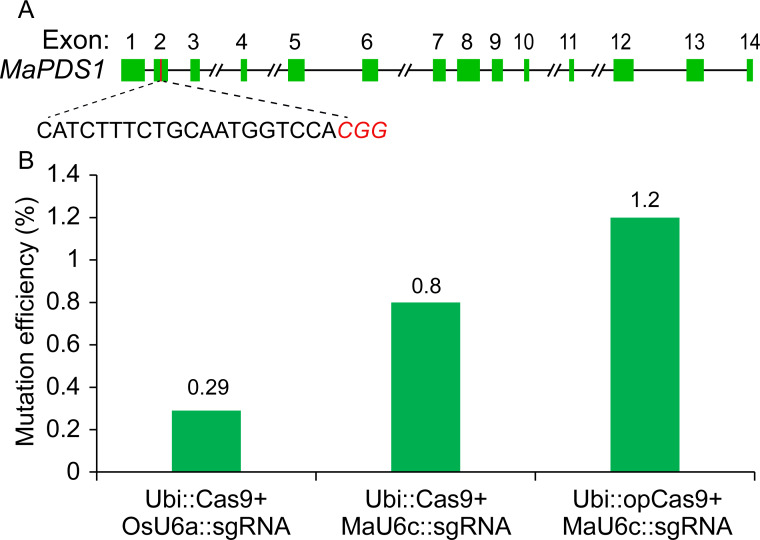
Validation of targeted mutagenesis of MaPDS1 induced by the optimized CRISPR/Cas9 system in banana. (A) Schematic position of the guide RNAs (red boxes) targeting the second exon of MaPDS. (B) Mutagenesis frequencies for PDS1 in ECS treated with different constructs revealed by deep amplicon sequencing. The experiment was repeated two times with similar results, and the result of one representative experiment was shown.

Given that the Cas9 used in our previous CRISPR/Cas9 system was Poaceae codon-optimized (Ma et al., 2015), we thought that the targeted genome editing efficiency could be further increased using banana codon-optimized Cas9 (Supplementary File 1). Therefore, we generated a new construct using banana codon-optimized Cas9 and pMaU6c (700 bp). To determine the activity of the new CRISPR/Cas9 system for banana endogenous gene editing, we selected one phytoene desaturase (*PDS*) target site. A banana protoplast transient assay was conducted to examine the functioning of the optimized CRISPR/Cas9 system. We isolated genomic DNA from the transformed protoplasts cultured for five days and used the amplicons that included target sites by two-round polymerase chain reaction (PCR) for deep sequencing. The results revealed that the replacement of the OsU6a promoter with MaU6c apparently improved the mutagenesis frequency of the PDS editing site (Fig. 4). The codon optimization of the Cas9 protein further increased the editing efficiency of the target site (Fig. 4, Supplementary File 4). Overall, the editing efficiency was increased by about fourfold by using a new genome editing construct in banana protoplasts.

### Generation of the frameshift in the fused-LUC (fLUC) system

**Figure 5:**
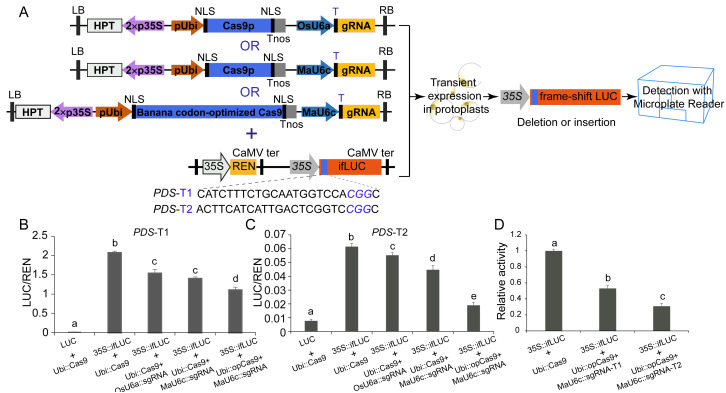
Generation of fLUC reporting system in banana. (A) Diagram of the fLUC reporting system. The 24 bp target sequence of sgRNA-MaPDS1 was fused after the LUC start codon to obtain fLUC (p35S::fLUC). The CRISPR/Cas9 expression cassette was co-expressed with p35S::fLUC in protoplasts. The p35S::fLUC was used as a control. (B–D) Examination of CRISPR/Cas9-mediated mutation efficiency using the fLUC system. Vertical bars represent standard deviations of the four independent biological replicates. Letters indicate significantly different values assessed using one-way analysis variance (ANOVA), followed by Fisher’s protected least significant difference test ( *P* < 0.05).

We used a luciferase reporter system to further compare the mutagenesis efficiency before and after optimization of CRISPR/Cas9 cassettes. In this system, a 24 bp sequence containing the sgRNA-PDS target site was inserted after the start codon of the LUC coding sequence, causing fused expression of the LUC protein. In addition, a 35 S promoter was introduced before the fused LUC coding sequence for driving the expression of the fused LUC protein (Figs. 5A, 5B, 5C). We observed apparent LUC activity in protoplasts co-transformed with the *p35S::fLUC* construct and Ubi::Cas9 plasmid (Figs. 5A, 5B, 5C), suggesting that the insertion of the 24 bp sequence did not affect the function of the LUC protein and that the coding sequence exhibited normal activity driven by the 35S promoter (Figs. 5A, 5B, 5C). CRISPR/Cas9-mediated genome editing usually caused a variety of indel mutations in the target site, some of which generated a frameshift in fused-*LUC* resulting in a nonfunctional LUC protein that could not be detected by enzymatic activity assays (Figs. 5A, 5B, 5C). The LUC activity of protoplasts co-transformed with *p35S::fLUC* and the functional CRISPR/Cas9 cassette was significantly decreased, suggesting impairment of coding sequences by indels caused by CRISPR/Cas9-mediated genome editing (Fig. 5). Moreover, the replacement of the OsU6a promoter with the MaU6c promoter or codon-optimized Cas9 in CRISPR/Cas9 cassette both decreased the LUC activity further in two targets of *MaPDS*, confirming that optimization of CRISPR/Cas9 cassette significantly improved mutagenesis efficiency in banana. We further compared the activity of two different sgRNAs from the same PDS target using this system. The results showed that PDS1-T2 had lower LUC activity than PDS-T1, suggesting the higher target activity of PDS1-T2 for genome editing of the PDS1 locus (Fig. 5D). These results indicated that the fused-LUC system was effective for validating the activity of different target sgRNAs before stable transformation.

## Discussion

Although CRISPR/Cas9 genome editing has now been widely used in many fruit crops, its application in banana is restricted by the difficulty of the genetic transformation system as well as by heterozygosity and polyploidy. At present, the most promising method for genetic transformation in banana is *Agrobacterium tumefaciens*–mediated transformation based on embryonic suspension cells (ECS), and this method is time consuming and labor intensive. Moreover, the triploid character of the predominant cultivated varieties makes it difficult for the gene-edited banana to eliminate integrated exogenous DNA by hybridization or selfing. The development of transgene-free gene editing technology is critical for the application of CRISPR/Cas9 genome editing in banana, because transgene-free mutant plants will be more acceptable and will reduce the concerns of biosecurity and ethics. The strategies of transgene-free gene editing in banana, however, usually need to skip the screening process of using a selective marker to reduce the integration of vector fragments (Chen et al., 2018), or directly deliver CRISPR/Cas9 ribonucleoprotein complex (RNPs) into receptor materials such as ECS (Svitashev et al., 2016). The common character of these approaches is that the function time of expressed sgRNA and Cas9 or preassembled RNPs in receptor materials is limited due to no integration of expression cassette or degradation. Furthermore, cultivated bananas are triploid, and it is very challenging to edit all allelic genes within a short time compare to diploid species. Therefore, the high mutation efficiency of the CRISPR/Cas9 genome editing system is important for the development of genome editing technology, especially for the establishment of a transgene-free gene editing system in banana.

The Cas9 nuclease and the single-guide RNA (sgRNA) are the two key components of the CRISPR/Cas9 genome editing system. In addition, increasing the expression levels of sgRNA and Cas9 proteins at the same time can significantly increase the incidence of targeted mutations in plants. The RNA polymerase III promoters, such as the U6 promoter, commonly are used for driving small RNAs expression in genome editing. Normally, the Arabidopsis U6 promoter and rice U6 promoter exhibit high activity for driving sgRNA expression in monocot and dicot plants, respectively (Ma et al., 2015). In our previous report, the combination of Poaceae codon-optimized Cas9 and sgRNA driven by the OsU6a promoter works in banana, but this method may be suboptimal (Hu et al., 2017a; Hu et al., 2017b). Recently, many reports have indicated that endogenous U6 promoters can increase editing efficiencies in different plant species (Sun et al., 2015; Long et al., 2018; Johansen et al., 2019). In this study, our results showed that endogenous U6 promoters were 1.3−2.2 times more active than OsU6a promoters in banana (Fig. 2). In addition, the banana U3 promoter also exhibited higher activity than that of the OsU6a promoter (data not shown). The high activity of endogenous promoters resulted in higher mutagenesis efficiency in banana protoplasts (Fig. 4). Although the expression level of Cas9 in some plants is considered to be a minor limiting factor for mutation efficiency, the application of banana codon-optimized Cas9 further improved the editing efficiency in our system (Fig. 4). Recently, LeBlanc et al. (2018) reported that 37 °C heat treatment can increase both cleavage activity of SpCas9 and efficiency of targeted mutagenesis *in vivo* in plants. For further increase the mutation efficiency, it is worth trying that adding heat treatment to the banana transformation protocol in our following study.

A recent report indicated that modifying the sgRNA structure dramatically improved knockout efficiency in TZM-bl cell lines (Dang et al., 2015). Therefore, optimization of sgRNA structure is another strategy to improve the editing efficiency in banana. Because the tRNA may function as a transcriptional enhancer for Pol III in plants (Xie, Minkenberg & Yang, 2015), we believe that a combination of tRNA and sgRNA will further increase the endogenous expression of gRNA *in vivo*. This may further improve the targeted mutagenesis efficiency of CRISPR/Cas9 in banana. Moreover, based on the endogenous tRNA processing, high-efficiency CRISPR/Cas9 multiplex gene editing systems have been developed in different plants (Xie, Minkenberg & Yang, 2015; Qi et al., 2016; Gasparis et al., 2018; Ma et al., 2019). This work has suggested that it is feasible for establishing a multiplex gene editing system in banana based on the polycistronic tRNA-gRNA (PTG) method.

The banana genetic transformation is a time- and labor-consuming process, and thus it is highly recommended to screen for high-activity sgRNA before attempting the stable transformation for genome editing. Usually, PCR-RE or amplicon sequencing are used to evaluate the different sgRNAs using protoplast transient transformation in plants, but a restriction enzyme site in the target sequence is required for PCR-RE assay and amplicon sequencing, which takes time and money. In this study, we developed a fused-LUC (fLUC) system for this purpose. This method quantitatively examined the genome editing efficiency of different sgRNAs and could be used in high-throughput systems with a microplate reader. It takes only several days from designing appropriate sgRNA targets to functional screening through this system, and the method can be applied in the validation of sgRNAs in other plant species.

## Conclusions

In this study, we reported that the endogenous U6 promoter and banana codon-optimized Cas9 apparently increased mutation frequency in banana. Moreover, we generated a method to validate the mutation efficiency of the CRISPR/Cas9-mediated genome editing system based on transient expression in protoplasts. This method could quantitatively examined the genome editing efficiency of different sgRNAs and could be used in high-throughput systems with a microplate reader before conducting stable genetics transformation.

## Supplemental Information

10.7717/peerj.12664/supp-1 Supplemental Information 1 Sequences of Banana codon-optimizated Cas9

10.7717/peerj.12664/supp-2 Supplemental Information 2 Sequences of promoter used in this study U6 promoters used in this study

10.7717/peerj.12664/supp-3 Supplemental Information 3 Primers used in this study

10.7717/peerj.12664/supp-4 Supplemental Information 4 Different types of targeted mutations The sgRNA target sequence is shown in figure. Deletions are shown as red dashes, and insertions are denoted with red letters and the mutation types on the right.

10.7717/peerj.12664/supp-5 Supplemental Information 5 Fig. 2 Raw Data Preliminary activity analysis of U6 promoter

10.7717/peerj.12664/supp-6 Supplemental Information 6 Fig. 3 Raw Data U6 promoter activity analysis

10.7717/peerj.12664/supp-7 Supplemental Information 7 Fig. 4 Raw Data

10.7717/peerj.12664/supp-8 Supplemental Information 8 Fig. 5 Raw Data

10.7717/peerj.12664/supp-9 Supplemental Information 9 Repeated experiment of Fig. 3

## Additional Information and Declarations

### Data Availability

The following information was supplied regarding data availability: The raw measurements are available in the Supplementary Files.

## References

[ref-1] Bernard G, Gagneul D, Alves Dos Santos H, Etienne A, Hilbert JL, Rambaud C (2019). Efficient genome editing using CRISPR/Cas9 technology in Chicory. International Journal of Molecular Sciences.

[ref-2] Bortesi L, Fischer R (2015). The CRISPR/Cas9 system for plant genome editing and beyond. Biotechnology Advances.

[ref-3] Bortesi L, Zhu C, Zischewski J, Perez L, Bassie L, Nadi R, Forni G, Lade SB, Soto E, Jin X, Medina V, Villorbina G, Munoz P, Farre G, Fischer R, Twyman RM, Capell T, Christou P, Schillberg S (2016). Patterns of CRISPR/Cas9 activity in plants, animals and microbes. Plant Biotechnol Journal.

[ref-4] Budman J, Chu G (2005). Processing of DNA for nonhomologous end-joining by cell-free extract. EMBO Journal.

[ref-5] Chandrasekaran J, Brumin M, Wolf D, Leibman D, Klap C, Pearlsman M, Sherman A, Arazi T, Gal-On A (2016). Development of broad virus resistance in non-transgenic cucumber using CRISPR/Cas9technology. Molecular Plant Pathology.

[ref-6] Cong L, Ran FA, Cox D, Lin S, Barretto R, Habib N, Hsu PD, Wu X, Jiang W, Marraffini LA, Zhang F (2013). Multiplex genome engineering using CRISPR/Cas systems. Science.

[ref-7] Dang Y, Jia G, Choi J, Ma H, Anaya E, Ye C, Shankar P, Wu H (2015). Optimizing sgRNA structure to improve CRISPR-Cas9 knockout efficiency. Genome Biology.

[ref-8] Dong T, Bi F-c, Huang Y-h, He W-d, Deng G-m, Gao H-j, Sheng O, Li C-y, Yang Q-s, Yi G-j, Hu C-h (2020). Highly efficient biolistic transformation of embryogenic cell suspensions of banana via a liquid medium selection system. Hortscience.

[ref-9] Eid A, Ali Z, Mahfouz MM (2016). High efficiency of targeted mutagenesis in arabidopsis via meiotic promoter-driven expression of Cas9 endonuclease. Plant Cell Reports.

[ref-10] Feng C, Su H, Bai H, Wang R, Liu Y, Guo X, Liu C, Zhang J, Yuan J, Birchler JA, Han F (2018). High-efficiency genome editing using a dmc1 promoter-controlled CRISPR/Cas9 system in maize. Plant Biotechnol Journal.

[ref-11] Feng J, Dai C, Luo H, Han Y, Liu Z, Kang C (2019). Reporter gene expression reveals precise auxin synthesis sites during fruit and root development in wild strawberry. Journal of Experimental Botany.

[ref-12] Gasparis S, Kala M, Przyborowski M, Lyznik LA, Orczyk W, Nadolska-Orczyk A (2018). A simple and efficient CRISPR/Cas9 platform for induction of single and multiple, heritable mutations in barley (Hordeum vulgare L.). Plant Methods.

[ref-13] Gong C, Bongiorno P, Martins A, Stephanou NC, Zhu H, Shuman S, Glickman MS (2005). Mechanism of nonhomologous end-joining in mycobacteria: a low-fidelity repair system driven by Ku, ligase D and ligase C. Nature Structural & Molecular Biology.

[ref-14] Hu B, Li D, Liu X, Qi J, Gao D, Zhao S, Huang S, Sun J, Yang L (2017a). Engineering non-transgenic gynoecious cucumber using an improved transformation protocol and optimized CRISPR/Cas9 system. Molecular Plant.

[ref-15] Hu C, Deng G, Sun X, Zuo C, Li C, Kuang R, Yang Q, Yi G (2017b). Establishment of an efficient CRISPR/Cas9-mediated gene editing system in banana. Scientia Agricultura Sinica.

[ref-16] Jia H, Orbovic V, Jones JB, Wang N (2016). Modification of the PthA4 effector binding elements in Type I CsLOB1 promoter using Cas9/sgRNA to produce transgenic Duncan grapefruit alleviating XccDeltapthA4:dCsLOB1.3 infection. Plant Biotechnol Journal.

[ref-17] Jia H, Wang N (2014). Targeted genome editing of sweet orange using Cas9/sgRNA. PLOS ONE.

[ref-18] Jia H, Zhang Y, Orbovic V, Xu J, White FF, Jones JB, Wang N (2017). Genome editing of the disease susceptibility gene CsLOB1 in citrus confers resistance to citrus canker. Plant Biotechnol Journal.

[ref-19] Jinek M, Chylinski K, Fonfara I, Hauer M, Doudna JA, Charpentier E (2012). A programmable dual-RNA-guided DNA endonuclease in adaptive bacterial immunity. Science.

[ref-20] Johansen IE, Liu Y, Jorgensen B, Bennett EP, Andreasson E, Nielsen KL, Blennow A, Petersen BL (2019). High efficacy full allelic CRISPR/Cas9 gene editing in tetraploid potato. Scientific Reports.

[ref-21] Kaur N, Alok A, Shivani Kaur N, Pandey P, Awasthi P, Tiwari S (2018). CRISPR/Cas9-mediated efficient editing in phytoene desaturase (PDS) demonstrates precise manipulation in banana cv. Rasthali genome. Functional & Integrative Genomics.

[ref-22] Kim YG, Cha J, Chandrasegaran S (1996). Hybrid restriction enzymes: zinc finger fusions to Fok I cleavage domain. Proceedings of the National Academy of Sciences of the United States of America.

[ref-23] Chen L-z, Li W, Katin-Grazzini L, Ding J, Gu X-b, Li Y-j, Gu T-t, Wang R, L X-c, Deng Z-n, McAvoy R, Gmitter Jr F, Deng Z-n, Zhao Y-d, Li Y (2018). A method for the production and expedient screening of CRISPR/Cas9-mediated non-transgenic mutant plants. Horticulture Research.

[ref-24] LeBlanc C, Zhang F, Mendez J, Lozano Y, Chatpar K, Irish VF, Jacob Y (2018). Increased efficiency of targeted mutagenesis by CRISPR/Cas9 in plants using heat stress. Plant Journal.

[ref-25] Li T, Yang X, Yu Y, Si X, Zhai X, Zhang H, Dong W, Gao C, Xu C (2018). Domestication of wild tomato is accelerated by genome editing. Nature Biotechnology.

[ref-26] Long L, Guo DD, Gao W, Yang WW, Hou LP, Ma XN, Miao YC, Botella JR, Song CP (2018). Optimization of CRISPR/Cas9 genome editing in cotton by improved sgRNA expression. Plant Methods.

[ref-27] Ma C, Zhu C, Zheng M, Liu M, Zhang D, Liu B, Li Q, Si J, Ren X, Song H (2019). CRISPR/Cas9-mediated multiple gene editing in Brassica oleracea var. capitata using the endogenous tRNA-processing system. Horticulture Research.

[ref-28] Ma X, Zhang Q, Zhu Q, Liu W, Chen Y, Qiu R, Wang B, Yang Z, Li H, Lin Y, Xie Y, Shen R, Chen S, Wang Z, Chen Y, Guo J, Chen L, Zhao X, Dong Z, Liu YG (2015). A Robust CRISPR/Cas9 system for convenient, high-efficiency multiplex genome editing in monocot and dicot Plants. Molecular Plant.

[ref-29] Mali P, Yang L, Esvelt KM, Aach J, Guell M, Di Carlo JE, Norville JE, Church GM (2013). RNA-guided human genome engineering via Cas9. Science.

[ref-30] Malnoy M, Viola R, Jung MH, Koo OJ, Kim S, Kim JS, Velasco R, Nagamangala Kanchiswamy C (2016). DNA-free genetically edited grapevine and apple protoplast using CRISPR/Cas9 ribonucleoproteins. Frontiers in Plant Science.

[ref-31] Mao Y, Botella JR, Zhu JK (2017). Heritability of targeted gene modifications induced by plant-optimized CRISPR systems. Cellular and Molecular Life Sciences.

[ref-32] Mao Y, Zhang Z, Feng Z, Wei P, Zhang H, Botella JR, Zhu JK (2016). Development of germ-line-specific CRISPR-Cas9 systems to improve the production of heritable gene modifications in Arabidopsis. Plant Biotechnol Journal.

[ref-33] Miller JC, Tan S, Qiao G, Barlow KA, Wang J, Xia DF, Meng X, Paschon DE, Leung E, Hinkley SJ, Dulay GP, Hua KL, Ankoudinova I, Cost GJ, Urnov FD, Zhang HS, Holmes MC, Zhang L, Gregory PD, Rebar EJ (2011). A TALE nuclease architecture for efficient genome editing. Nature Biotechnology.

[ref-34] Naim F, Dugdale B, Kleidon J, Brinin A, Shand K, Waterhouse P, Dale J (2018). Gene editing the phytoene desaturase alleles of Cavendish banana using CRISPR/Cas9. Transgenic Research.

[ref-35] Ng H, Dean N (2017). Dramatic Improvement of CRISPR/Cas9 Editing in Candida albicans by increased single guide RNA expression. MSphere.

[ref-36] Nishitani C, Hirai N, Komori S, Wada M, Okada K, Osakabe K, Yamamoto T, Osakabe Y (2016). Efficient genome editing in apple using a CRISPR/Cas9 system. Scientific Reports.

[ref-37] Ntui VO, Tripathi JN, Tripathi L (2020). Robust CRISPR/Cas9 mediated genome editing tool for banana and plantain (Musa spp.). Current Plant Biology.

[ref-38] Ordon J, Bressan M, Kretschmer C, Dall’Osto L, Marillonnet S, Bassi R, Stuttmann J (2020). Optimized Cas9 expression systems for highly efficient Arabidopsis genome editing facilitate isolation of complex alleles in a single generation. Functional & Integrative Genomics.

[ref-39] Peng A, Chen S, Lei T, Xu L, He Y, Wu L, Yao L, Zou X (2017). Engineering canker-resistant plants through CRISPR/Cas9-targeted editing of the susceptibility gene CsLOB1 promoter in citrus. Plant Biotechnol Journal.

[ref-40] Qi W, Zhu T, Tian Z, Li C, Zhang W, Song R (2016). High-efficiency CRISPR/Cas9 multiplex gene editing using the glycine tRNA-processing system-based strategy in maize. BMC Biotechnology.

[ref-41] Razzaq A, Saleem F, Kanwal M, Mustafa G, Yousaf S, Imran Arshad HM, Hameed MK, Khan MS, Joyia FA (2019). Modern trends in plant genome editing: an inclusive review of the CRISPR/Cas9 toolbox. International Journal of Molecular Sciences.

[ref-42] Ren C, Liu X, Zhang Z, Wang Y, Duan W, Li S, Liang Z (2016). CRISPR/Cas9-mediated efficient targeted mutagenesis in Chardonnay (Vitis vinifera L.). Scientific Reports.

[ref-43] Simmonds NW, Shepherd K (1955). The taxonomy and origins of the cultivated bananas. Botanical Journal of the Linnean Society.

[ref-44] Sun X, Hu Z, Chen R, Jiang Q, Song G, Zhang H, Xi Y (2015). Targeted mutagenesis in soybean using the CRISPR-Cas9 system. Scientific Reports.

[ref-45] Svitashev S, Schwartz C, Lenderts B, Young J-k, Cigan A-m (2016). Genome editing in maize directed by CRISPR–Cas9 ribonucleoprotein complexes. Nature Communications.

[ref-46] Tian S, Jiang L, Gao Q, Zhang J, Zong M, Zhang H, Ren Y, Guo S, Gong G, Liu F, Xu Y (2017). Efficient CRISPR/Cas9-based gene knockout in watermelon. Plant Cell Reports.

[ref-47] Tripathi JN, Ntui VO, Ron M, Muiruri SK, Britt A, Tripathi L (2019). CRISPR/Cas9 editing of endogenous banana streak virus in the B genome of Musa spp. overcomes a major challenge in banana breeding. Communications Biology.

[ref-48] Tripathi L, Ntui VO, Tripathi JN (2020). CRISPR/Cas9-based genome editing of banana for disease resistance. Current Opinion in Plant Biology.

[ref-49] Wang X, Tu M, Wang D, Liu J, Li Y, Li Z, Wang Y, Wang X (2018a). CRISPR/Cas9-mediated efficient targeted mutagenesis in grape in the first generation. Plant Biotechnol Journal.

[ref-50] Wang Z, Miao H, Liu J, Xu B, Yao X, Xu C, Zhao S, Fang X, Jia C, Wang J, Zhang J, Li J, Xu Y, Wang J, Ma W, Wu Z, Yu L, Yang Y, Liu C, Guo Y, Sun S, Baurens FC, Martin G, Salmon F, Garsmeur O, Yahiaoui N, Hervouet C, Rouard M, Laboureau N, Habas R, Ricci S, Peng M, Guo A, Xie J, Li Y, Ding Z, Yan Y, Tie W, D’Hont A, Hu W, Jin Z (2019). Musa balbisiana genome reveals subgenome evolution and functional divergence. Nature Plants.

[ref-51] Wang Z, Wang S, Li D, Zhang Q, Li L, Zhong C, Liu Y, Huang H (2018b). Optimized paired-sgRNA/Cas9 cloning and expression cassette triggers high-efficiency multiplex genome editing in kiwifruit. Plant Biotechnol Journal.

[ref-52] Wang ZP, Xing HL, Dong L, Zhang HY, Han CY, Wang XC, Chen QJ (2015). Egg cell-specific promoter-controlled CRISPR/Cas9 efficiently generates homozygous mutants for multiple target genes in Arabidopsis in a single generation. Genome Biology.

[ref-53] Wu S, Zhu H, Liu J, Yang Q, Shao X, Bi F, Hu C, Huo H, Chen K, Yi G (2020). Establishment of a PEG-mediated protoplast transformation system based on DNA and CRISPR/Cas9 ribonucleoprotein complexes for banana. BMC Plant Biology.

[ref-54] Xie K, Minkenberg B, Yang Y (2015). Boosting CRISPR/Cas9 multiplex editing capability with the endogenous tRNA-processing system. Proceedings of the National Academy of Sciences of the United States of America.

[ref-55] Yan L, Wei S, Wu Y, Hu R, Li H, Yang W, Xie Q (2015). High-efficiency genome editing in Arabidopsis using YAO promoter-driven CRISPR/Cas9 system. Molecular Plant.

[ref-56] Yang Y, Zhu G, Li R, Yan S, Fu D, Zhu B, Tian H, Luo Y, Zhu H (2017). The RNA editing factor SlORRM4 Is required for normal fruit ripening in tomato. Plant Physiology.

[ref-57] Yu QH, Wang B, Li N, Tang Y, Yang S, Yang T, Xu J, Guo C, Yan P, Wang Q, Asmutola P (2017). CRISPR/Cas9-induced targeted mutagenesis and gene replacement to generate long-shelf life tomato lines. Scientific Reports.

[ref-58] Zha S, Boboila C, Alt FW (2009). Mre11: roles in DNA repair beyond homologous recombination. Nature Structural & Molecular Biology.

[ref-59] Zhang F, LeBlanc C, Irish VF, Jacob Y (2017). Rapid and efficient CRISPR/Cas9 gene editing in Citrus using the YAO promoter. Plant Cell Reports.

[ref-60] Zhou J, Wang G, Liu Z (2018). Efficient genome editing of wild strawberry genes, vector development and validation. Plant Biotechnol Journal.

[ref-61] Zhu J, Song N, Sun S, Yang W, Zhao H, Song W, Lai J (2016). Efficiency and inheritance of targeted mutagenesis in maize using CRISPR-Cas9. Journal of Genetics and Genomics.

