# Supplementary material for: Increased mutation efficiency of CRISPR/Cas9 genome editing in banana by optimized construct
Source: PeerJ. 2022 Jan 5;10:e12664. doi: 10.7717/peerj.12664 (PMC8742547; doi:10.7717/peerj.12664)
Supplement: Supplemental Information 1 [file peerj-10-12664-s001.docx]

**>** ***Banana codon-optimizated Cas9***

ATGGCACCGAAAAAAAAGAGGAAGGTCGGTATACATGGGGTTCCTGCAGCTGATAAAAAGTATAGCATTGGCTTGGACATAGGGACTAATTCTGTTGGTTGGGCCGTCATAACAGATGAGTACAAAGTACCTTCTAAGAAGTTCAAGGTTCTGGGTAACACCGACCGTCACTCTATTAAGAAGAACCTCATTGGCGCTCTTCTGTTCGACTCCGGGGAGACCGCTGAAGCAACGCGGTTGAAGCGTACCGCTCGGCGGCGTTATACCAGGCGTAAGAACCGTATTTGCTACTTGCAGGAGATATTCTCAAATGAGATGGCGAAAGTGGATGACTCTTTCTTCCATAGGTTGGAGGAGAGTTTCCTCGTTGAGGAAGATAAGAAACATGAGCGACATCCGATCTTCGGGAATATCGTTGATGAAGTCGCTTATCACGAGAAGTATCCCACGATCTACCATCTGCGGAAAAAGCTGGTCGATAGTACGGATAAGGCGGACTTGCGCCTCATCTACCTGGCTCTTGCACACATGATTAAGTTTAGGGGGCATTTCCTTATAGAAGGAGACCTTAACCCCGATAATAGTGACGTGGATAAATTGTTTATACAACTCGTTCAGACGTACAACCAATTGTTCGAGGAGAACCCAATAAATGCAAGTGGTGTCGATGCTAAAGCAATTTTGTCAGCGAGACTCTCCAAGAGCCGTCGTCTCGAAAACCTTATAGCTCAACTGCCCGGGGAGAAAAAAAACGGGTTGTTCGGCAACCTCATTGCGTTGTCTTTGGGCCTGACTCCGAATTTTAAATCAAATTTCGACCTCGCAGAGGACGCCAAGCTCCAACTCAGTAAAGACACGTACGATGACGATCTCGACAATCTGCTGGCCCAGATTGGCGATCAATATGCCGATCTCTTTCTCGCAGCAAAGAATCTGTCTGACGCTATCCTGCTCTCAGACATCCTGAGAGTTAATACTGAGATCACGAAAGCACCTTTGTCAGCTAGTATGATAAAGCGTTATGATGAACATCACCAGGATCTGACCTTGCTTAAGGCCCTTGTCCGGCAGCAACTCCCTGAGAAATATAAGGAGATCTTCTTCGATCAATCCAAGAACGGGTATGCTGGGTATATCGATGGGGGCGCTTCACAGGAAGAGTTCTACAAGTTTATCAAACCTATTTTGGAGAAGATGGACGGAACGGAGGAACTGCTCGTCAAGCTTAACCGTGAGGATCTTCTCCGAAAACAGAGAACATTCGATAATGGCTCAATACCACATCAAATCCATCTCGGGGAGCTTCACGCAATCCTCCGACGGCAAGAGGACTTCTACCCATTCTTGAAGGATAATCGTGAGAAGATTGAAAAGATTTTGACATTCCGGATACCATATTATGTGGGGCCACTGGCCCGCGGTAATTCTCGCTTCGCCTGGATGACCCGGAAGTCCGAGGAGACTATCACGCCGTGGAATTTTGAAGAGGTTGTTGATAAGGGGGCTTCGGCCCAGTCATTCATAGAGAGGATGACGAATTTCGACAAGAACTTGCCAAATGAGAAAGTGCTCCCTAAGCATAGTTTGTTGTATGAGTATTTCACAGTGTACAATGAACTCACTAAAGTAAAGTACGTTACGGAAGGCATGCGCAAGCCTGCCTTTCTCTCGGGTGAGCAAAAAAAGGCTATCGTCGATCTGTTGTTTAAGACTAATCGGAAGGTCACAGTCAAGCAACTGAAGGAGGACTACTTTAAAAAAATTGAGTGCTTCGACTCAGTGGAGATTAGCGGGGTGGAAGATAGGTTCAATGCGTCCTTGGGCACTTACCACGACTTGCTGAAGATCATCAAGGACAAAGATTTTCTTGACAATGAAGAAAACGAAGATATTTTGGAGGATATCGTCTTGACTCTGACCCTGTTTGAGGATAGGGAAATGATTGAGGAAAGACTGAAGACGTACGCGCATTTGTTTGATGACAAGGTGATGAAGCAGTTGAAAAGACGGCGGTATACTGGATGGGGGAGGCTCTCTAGAAAGTTGATTAACGGTATCAGAGATAAACAGAGTGGCAAGACCATACTCGATTTTCTCAAATCAGACGGATTCGCTAATAGGAATTTCATGCAGCTGATCCATGACGACTCTCTCACCTTCAAAGAGGACATTCAGAAAGCCCAAGTTTCAGGACAGGGTGACAGTCTCCACGAACATATCGCAAACCTCGCTGGCTCTCCCGCTATTAAGAAAGGGATTCTGCAAACTGTAAAGGTGGTCGATGAGCTTGTGAAAGTTATGGGAAGGCATAAGCCTGAGAACATTGTGATTGAAATGGCGAGGGAGAACCAAACTACTCAGAAGGGTCAAAAAAACTCGAGAGAGCGTATGAAGCGAATTGAGGAGGGCATCAAGGAGTTGGGCTCTCAGATATTGAAGGAGCACCCAGTGGAGAATACCCAATTGCAAAATGAGAAGCTGTATCTCTACTACCTTCAGAATGGAAGGGATATGTACGTGGACCAAGAACTGGATATTAATCGGCTCTCGGATTACGATGTTGACCATATTGTTCCGCAGTCATTCCTCAAAGATGACAGTATTGATAATAAAGTGCTTACCCGTAGCGATAAGAATAGGGGAAAATCCGACAACGTGCCAAGTGAGGAGGTGGTGAAAAAGATGAAGAACTATTGGCGTCAGCTCCTGAATGCAAAGCTTATAACACAGCGTAAATTCGACAACCTGACCAAGGCTGAGCGTGGTGGGCTCTCAGAACTCGATAAGGCGGGGTTCATTAAACGGCAGCTCGTAGAGACTCGGCAGATCACCAAACACGTGGCACAAATCCTGGACTCTAGGATGAACACCAAGTATGACGAGAATGACAAGCTGATTCGTGAAGTCAAGGTTATTACCCTCAAGAGCAAGTTGGTCTCAGATTTTAGGAAAGATTTTCAGTTCTACAAAGTTCGCGAGATCAATAACTATCACCATGCACACGATGCATACCTGAATGCCGTCGTTGGGACAGCCCTGATCAAAAAGTACCCTAAGCTGGAGTCCGAGTTTGTGTACGGAGACTACAAGGTGTACGATGTAAGGAAAATGATCGCGAAGTCTGAGCAAGAGATAGGCAAGGCAACTGCAAAGTATTTCTTCTACTCTAATATAATGAATTTCTTTAAGACCGAGATCACGCTTGCCAATGGCGAGATTAGGAAGAGACCCCTGATAGAGACTAACGGGGAAACGGGTGAGATTGTTTGGGATAAAGGGCGGGATTTCGCGACGGTTCGGAAGGTGTTGTCTATGCCTCAGGTTAACATAGTTAAGAAGACTGAGGTCCAGACGGGTGGATTCTCAAAGGAGAGCATCCTGCCTAAACGTAATAGTGACAAATTGATAGCACGGAAGAAGGATTGGGACCCTAAGAAGTACGGCGGATTCGATTCTCCGACCGTAGCCTACAGTGTTCTGGTGGTGGCCAAGGTCGAGAAGGGAAAGAGCAAGAAGCTGAAATCCGTGAAGGAACTGTTGGGGATAACTATAATGGAGCGTAGTTCGTTTGAAAAGAACCCTATTGATTTCCTTGAAGCCAAGGGTTACAAAGAAGTGAAGAAGGATCTGATCATCAAGCTTCCCAAGTACTCACTGTTTGAGCTGGAGAACGGAAGGAAAAGGATGTTGGCATCCGCTGGTGAGCTCCAGAAGGGGAATGAGCTCGCTTTGCCTAGTAAGTACGTGAATTTCCTCTACCTCGCCTCACACTATGAAAAGCTGAAGGGATCACCGGAAGACAATGAGCAGAAGCAACTCTTTGTGGAACAACACAAGCACTACTTGGATGAGATAATTGAGCAAATTTCAGAGTTTAGCAAAAGAGTGATTTTGGCAGACGCTAACCTGGACAAAGTCTTGTCCGCATATAATAAGCACCGGGACAAACCAATCCGTGAGCAAGCCGAGAACATTATACATTTGTTTACCCTTACTAACCTCGGCGCACCGGCAGCATTTAAGTATTTCGACACGACCATAGATAGAAAACGTTACACCTCAACAAAGGAAGTGCTGGACGCTACTCTCATTCACCAATCGATTACTGGCCTTTATGAGACAAGAATTGACCTCTCTCAGTTGGGCGGCGACAAAAGGCCGGCTGCTACAAAGAAAGCTGGTCAAGCGAAGAAAAAGAAGTAA

**>** ***Banana codon-optimizated Cas9***

MAPKKKRKVGIHGVPAADKKYSIGLDIGTNSVGWAVITDEYKVPSKKFKVLGNTDRHSIKKNLIGALLFDSGETAEATRLKRTARRRYTRRKNRICYLQEIFSNEMAKVDDSFFHRLEESFLVEEDKKHERHPIFGNIVDEVAYHEKYPTIYHLRKKLVDSTDKADLRLIYLALAHMIKFRGHFLIEGDLNPDNSDVDKLFIQLVQTYNQLFEENPINASGVDAKAILSARLSKSRRLENLIAQLPGEKKNGLFGNLIALSLGLTPNFKSNFDLAEDAKLQLSKDTYDDDLDNLLAQIGDQYADLFLAAKNLSDAILLSDILRVNTEITKAPLSASMIKRYDEHHQDLTLLKALVRQQLPEKYKEIFFDQSKNGYAGYIDGGASQEEFYKFIKPILEKMDGTEELLVKLNREDLLRKQRTFDNGSIPHQIHLGELHAILRRQEDFYPFLKDNREKIEKILTFRIPYYVGPLARGNSRFAWMTRKSEETITPWNFEEVVDKGASAQSFIERMTNFDKNLPNEKVLPKHSLLYEYFTVYNELTKVKYVTEGMRKPAFLSGEQKKAIVDLLFKTNRKVTVKQLKEDYFKKIECFDSVEISGVEDRFNASLGTYHDLLKIIKDKDFLDNEENEDILEDIVLTLTLFEDREMIEERLKTYAHLFDDKVMKQLKRRRYTGWGRLSRKLINGIRDKQSGKTILDFLKSDGFANRNFMQLIHDDSLTFKEDIQKAQVSGQGDSLHEHIANLAGSPAIKKGILQTVKVVDELVKVMGRHKPENIVIEMARENQTTQKGQKNSRERMKRIEEGIKELGSQILKEHPVENTQLQNEKLYLYYLQNGRDMYVDQELDINRLSDYDVDHIVPQSFLKDDSIDNKVLTRSDKNRGKSDNVPSEEVVKKMKNYWRQLLNAKLITQRKFDNLTKAERGGLSELDKAGFIKRQLVETRQITKHVAQILDSRMNTKYDENDKLIREVKVITLKSKLVSDFRKDFQFYKVREINNYHHAHDAYLNAVVGTALIKKYPKLESEFVYGDYKVYDVRKMIAKSEQEIGKATAKYFFYSNIMNFFKTEITLANGEIRKRPLIETNGETGEIVWDKGRDFATVRKVLSMPQVNIVKKTEVQTGGFSKESILPKRNSDKLIARKKDWDPKKYGGFDSPTVAYSVLVVAKVEKGKSKKLKSVKELLGITIMERSSFEKNPIDFLEAKGYKEVKKDLIIKLPKYSLFELENGRKRMLASAGELQKGNELALPSKYVNFLYLASHYEKLKGSPEDNEQKQLFVEQHKHYLDEIIEQISEFSKRVILADANLDKVLSAYNKHRDKPIREQAENIIHLFTLTNLGAPAAFKYFDTTIDRKRYTSTKEVLDATLIHQSITGLYETRIDLSQLGGDKRPAATKKAGQAKKKK
