# Supplementary material for: Increased mutation efficiency of CRISPR/Cas9 genome editing in banana by optimized construct
Source: PeerJ. 2022 Jan 5;10:e12664. doi: 10.7717/peerj.12664 (PMC8742547; doi:10.7717/peerj.12664)
Supplement: Supplemental Information 4 — The sgRNA target sequence is shown in figure. Deletions are shown as red dashes, and insertions are denoted with red letters and the mutation types on the right. [file peerj-10-12664-s004.docx]

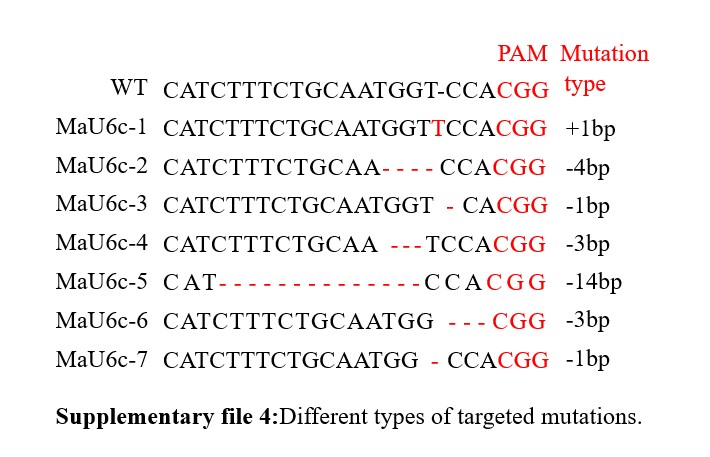


The sgRNA target sequence is shown in figure. Deletions are shown as red dashes, and insertions are denoted with red letters and the mutation types on the right.
